# Accuracy of preoperative tumor localization in large bowel using 3D magnetic endoscopic imaging: randomized clinical trial

**DOI:** 10.1007/s00464-016-5203-4

**Published:** 2016-08-29

**Authors:** Miroslaw Szura, Artur Pasternak, Rafal Solecki, Maciej Matyja, Antoni Szczepanik, Andrzej Matyja

**Affiliations:** 1 Department of Experimental and Clinical Surgery, Jagiellonian University Medical College, 12 Michalowskiego St., 31-126 Kraków, Poland; 2 First Chair of General, Oncological and Gastrointestinal Surgery, Jagiellonian University Medical College, 40th Kopernika St., 31-501 Kraków, Poland; 3 Department of Anatomy, Jagiellonian University Medical College, 12th Kopernika St., 31-034 Kraków, Poland; 4 2nd Chair of General Surgery, Jagiellonian University Medical College, 21st Kopernika St., 31-501 Kraków, Poland

**Keywords:** Colonoscopy, Colorectal cancer, Magnetic positioning system, Scope Guide

## Abstract

**Background:**

Laparoscopic surgery has become the standard treatment for colorectal cancer. A tumor that does not involve serosa is invisible intraoperatively, and manual palpation of the tumor during laparoscopy is not possible. Therefore, accurate localization of the neoplastic infiltrate remains one of the most important tasks prior to elective laparoscopic surgery. The aim of this study was to evaluate the utility of a magnetic endoscopic imaging (MEI) for precise preoperative endoscopic localization of neoplastic infiltrate within the large bowel.

**Materials and methods:**

The study enrolled 246 patients who underwent elective surgery for colorectal cancer in 2012–2015 with accurate preoperative colonoscopic localization of the tumor. The analysis concerned patients with neoplastic infiltrate localized more than 30 cm from the anal verge. For evaluative purposes and accuracy of localization, the intestine was divided anatomically into 13 parts. Colonoscopic examinations were conducted with two types of endoscopes: group I—with MEI and group II—without MEI. Patients were assigned to the groups by random allocation. Ultimate confirmation of the tumor localization was accomplished by intraoperative evaluation.

**Results:**

Group I involved 127 patients and group II 129. The two groups were compared in terms of age, sex, BMI and frequency of previous abdominal procedures. Proper localization of the lesion was confirmed in 95.23 % of group I patients and in 83.19 % of group II patients (*p* < 0.05). The greatest discrepancy in localization occurred in 8.9 % of patients from group I and 20 % of patients from group II in which the lesion was assessed primarily in the distal sigmoid.

**Conclusions:**

A magnetic endoscopic imaging allows more accurate localization of neoplastic infiltrate within the large intestine compared to standard colonoscopy alone, especially within the sigmoid colon. This method can be particularly useful in planning and performing laparoscopic procedures to diminish the likelihood of improper bowel segment resection.

**ClinicalTrials.gov number:**

NCT01688557

**Electronic supplementary material:**

The online version of this article (doi:10.1007/s00464-016-5203-4) contains supplementary material, which is available to authorized users.

Over the past 20 years, with the continuous development of laparoscopic surgical techniques and the invention and perfection of all types of laparoscopic equipment, laparoscopic colorectal surgery has gained encouraging achievements, and both its short- and long-term effects have been proved. The localization of a tumor may be critical in laparoscopic colorectal surgery because its manual palpation may not be possible. Moreover, tumors without serosal involvement are frequently laparoscopically invisible. Therefore, accurate preoperative identification of the tumor site remains one of the most important tasks preceding laparoscopic surgery. One method for overcoming these limitations is to provide a real-time view of the colonoscope position during examination especially when the colon tumor is detected. It has become feasible with use of magnetic endoscopic imaging (MEI, Scope Guide, Olympus Optical Co., Ltd.). This system provides continuous three-dimensional (3D) view of the scope shaft configuration and its location within the abdomen during colonoscopic examination [1]. The MEI system is composed of three basic elements: a graphics processor, the endoscope and a signal receiver (Figs. 1, 2). Positioned at regular intervals within the endoscope, along its entire length, are magnetic coils that constitute a generator, each of which generates a pulsed low-voltage magnetic field. The generator is connected to the endoscope through an attachment within it made just for that purpose. The magnetic signal is collected by an external-to-the-patient signal receiver, and the signal is then converted electronically to a 3D image on the screen [2]. The effect of spatial visualization is achieved by electronic processing, resulting in the position of the endoscope being shown in shades of gray as well as the topographical location of the tip of the endoscope and its exact location in relation to the abdominal wall. Software assesses the three-dimensional position and orientation of each receiver coil, and the data are displayed in real time as a computer-rendered 3D image of the colonoscope shaft configuration. The scope position can be displayed either in anteroposterior (AP) view alone or in split-screen view, which combines the AP and lateral views side by side. The split-screen view helps clarify the colonoscope loop configuration in 3D. MEI system has been shown to be beneficial in the localization of the colonoscope tip, which may be important for confirming cecal intubation and precise pathologic lesion localization (Video 1). The aim of this prospective study was to evaluate the usability of MEI for accurate preoperative endoscopic localization of neoplastic tumors within the large intestine.

**Fig. 1:**
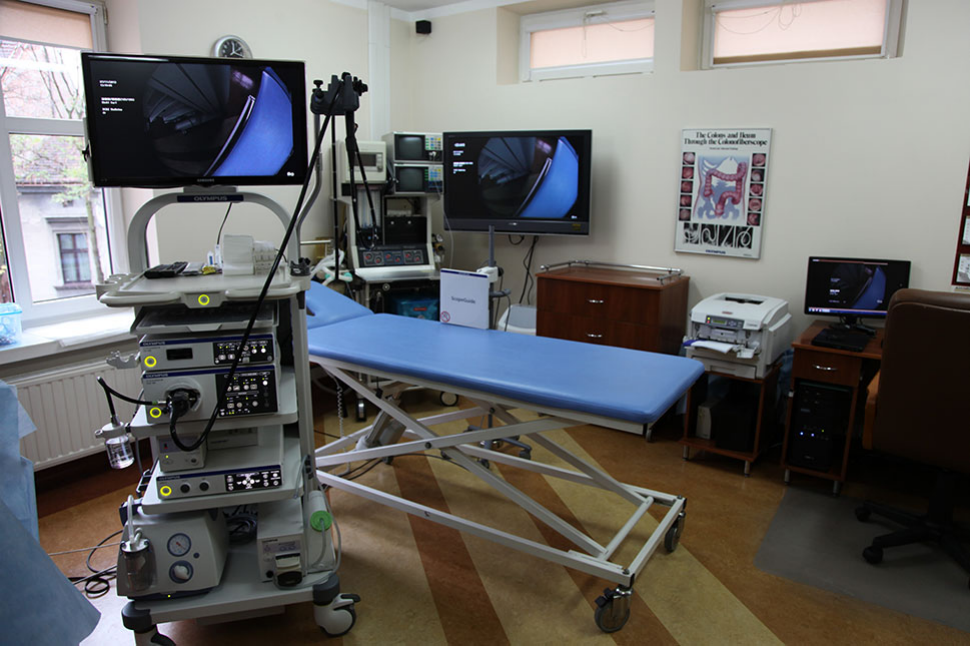
Position detecting unit integrated in the EVIS EXERA III system

**Fig. 2:**
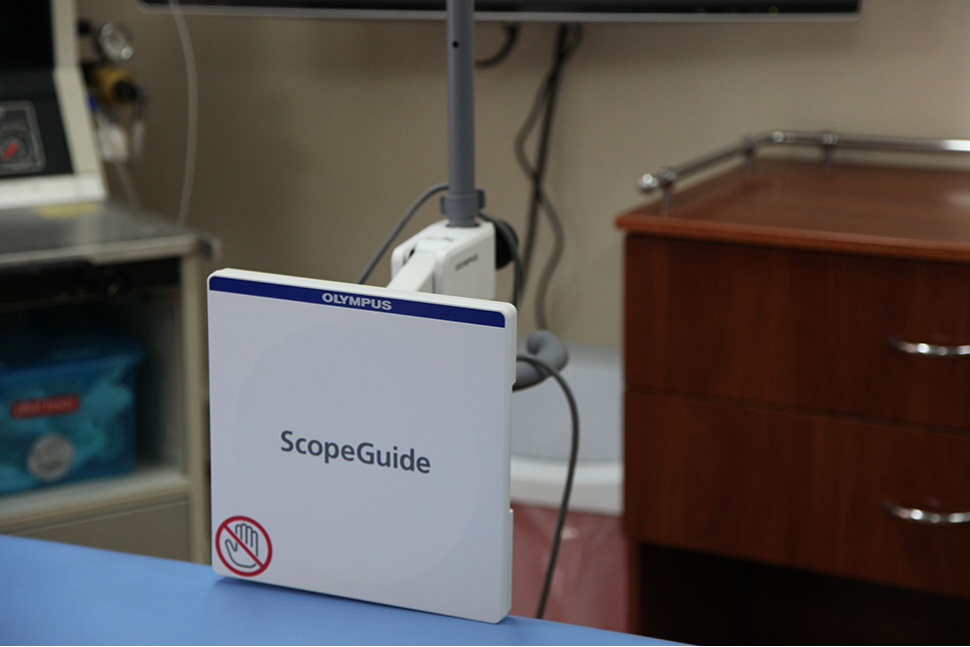
Electromagnetic receiver. The new Scope Guide receiver dish is compact and thin

## Materials and methods

**Fig. 3:**
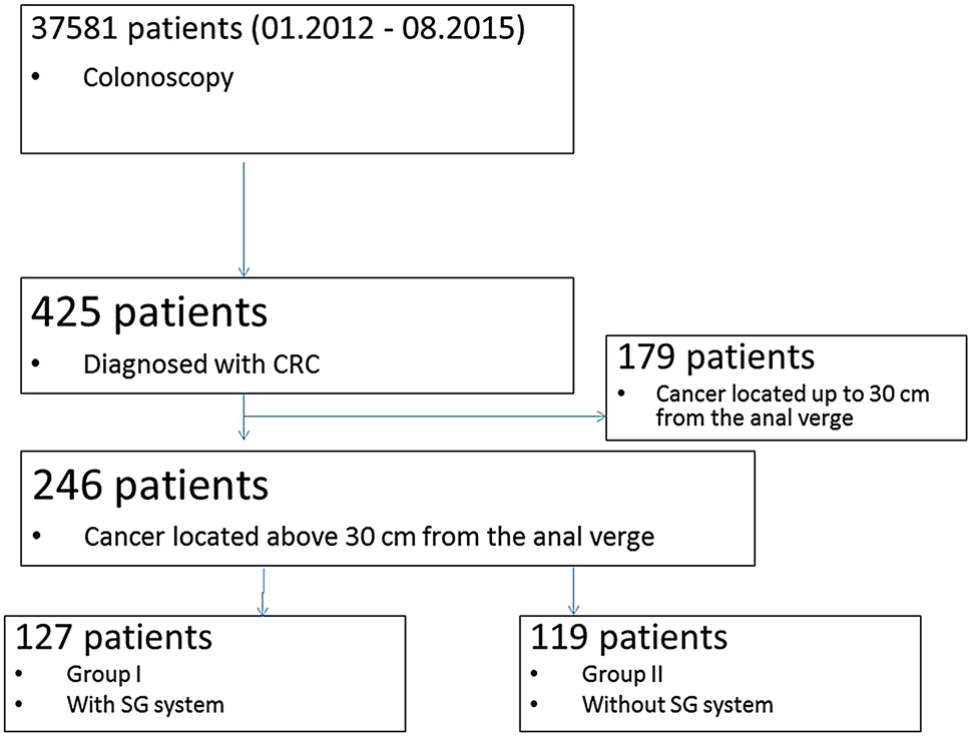
CONSORT diagram of patient enrollment

A total of 37 581 patients underwent colonoscopies between January 2012 and August 2015 (Fig. 3). The study included 425 patients who were diagnosed with colon cancer and treated surgically. One hundred and seventy-nine patients in whom the tumor infiltrate was located less than 30 cm from the anal verge were excluded from the study. The exclusion of these patients from the analysis was dictated by the fact that differences in the position of cancer in this section of the bowel do not affect the change of operating tactics. Finally, the analysis enrolled 246 patients, who were assigned to the groups by random allocation. Group I consisted of 127 patients who underwent colonoscopy with the use of MEI, and group II consisted of 119 patients who were examined without the use of this positioning device. Randomization was based on a random selection of the endoscope (equipped with MEI or not) to the individual patient. All the investigating physicians had appropriate certificates of know-how required by law, and possessed experience in the execution of more than 500 colonoscopies by each of them. As doctors had appropriate qualifications, it was assumed that their skills are comparable. Thus, their participation in the individual colonoscopic examinations was not randomized. The study protocol was approved by the local ethics committee and registered at ClinicalTrials.gov (Identification number: NCT01688557). The study was reported in accordance with the CONSORT statement.

Bowel preparation for colonoscopy was based solely on the oral ingestion of liquid propulsive agents, 420 grams of macrogolum in 4 L of water, to be exact, given in four doses every 6 h, 1 day preceding the colonoscopy.

**Fig. 4:**
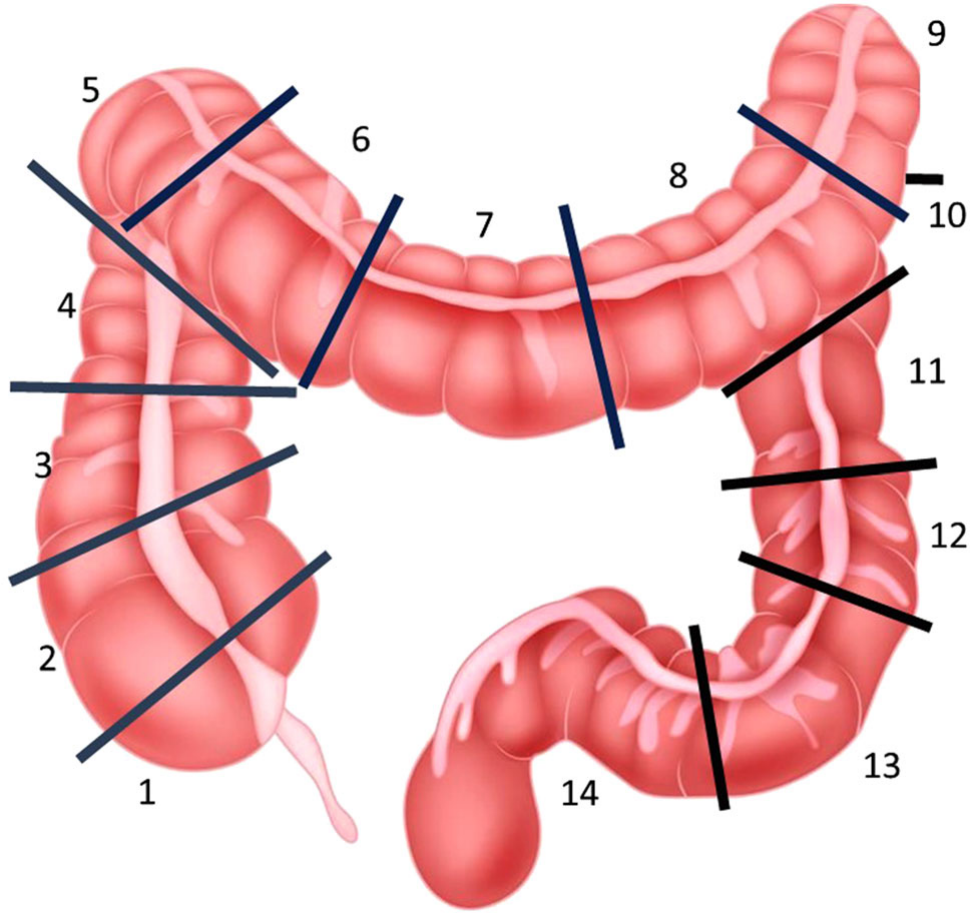
Sections of the colon for the exact localization of cancer site

We used Olympus series 180 and 190 instruments based on the high-definition technology HDTV 1080i. The examination was initiated with patient positioning in the left lateral position, but later on, the arrangement was changed as necessary. The location of the tumor in patients examined with MEI-equipped colonoscopes was determined on the basis of the image obtained using this system showing the exact position of the tip of the endoscope apposed in the direct proximity of the tumor margin within the intestine. In patients examined with standard colonoscopies not equipped with MEI, the location of the tumor was determined by the following elements: the characteristic endoscopic image of the involved bowel segment, the range of the scope inserted and by applying manual pressure to abdomen to localize the position of the tip of the endoscope within the intestine. Furthermore, for evaluative purposes and localization accuracy, each of the anatomic sections of large intestine was further divided into three parts. Eventually, 14 parts of the bowel were obtained; the last episode involving the rectum and sigmoid colon to a depth of 30 cm from the anal verge was excluded from further analysis (Fig. 4). Colonoscopists were advised to judiciously allocate the position of the tumor to adequate part of the bowel. Patients diagnosed with colon cancer underwent imaging and laboratory tests and were scheduled for elective surgery. Surgical bowel resections were performed laparoscopically or with open surgery. Qualification for laparoscopic or open surgery depended on the stage (extent) of the cancer, patient and surgeon preference and experience. During laparotomy, the tumor site was confirmed macroscopically and palpably, while in laparoscopy the tumor was localized macroscopically or by performing intraoperative colonoscopy owing to nonvisualization. The tumor site was also allocated according to the previously mentioned 14-segment scale. We did not analyze the advancement stage of the tumor because it was not a subject of this study, whereas we only assessed the accuracy of preoperative localization of the lesion in the intestine.

All data were prospectively collected and entered into the Access 2010 software and then transferred to the STATISTICA 12.0 software. The materials acquired in this study were systematized and analyzed, and a distribution of variables was established. Because the analyzed parameters do not have normal distribution, nonparametric tests were applied in the analysis. Qualitative variables were compared using the independent Chi-square test. For the comparison of quantitative variables, the Mann–Whitney test was used in two groups. Comparison of quantitative data in more than two groups was done by Kruskal–Wallis test. The materiality threshold was established at *p* ≤ 0.05.

## Results

Patients who were included in this study underwent surgical removal of the tumor with respect to the principles of oncologic resection. All the tumors were correctly localized intraoperatively, and there was no incorrect colonic segment resection.

To assess parameters that could be associated with errant preoperative tumor localization, we compared the frequency of erroneous endoscopic diagnoses with regard to the following variables: sex, age and body mass index. Hence, both groups were comparable in terms of age, sex and BMI. The overall characteristics are shown in Table 1.

**Table 1:** Characteristics of the groups

Group	Sex	*N*	Age min	Age max	Age mean		Age SD		BMI min	BMI max	BMI mean		BMI SD	
I	F	58	41	83	67.07	66.15	10.62	12.13	19	40	24.95	24.78	3.79	3.67
	M	69	27	87	65.38		13.29		19	34	24.64		3.58	
II	F	56	30	90	63.25	65.20	10.17	10.79	19	32	24.66	24.97	3.61	3.94
	M	63	42	89	66.94		11.11		19	39	25.24		4.23	

Sex *p* = 0.65; Age *p* = 0.519; BMI *p* = 0.7

Endoscopic tumor localization was accurate in the 121 group I patients (95.28 %) in whom the MEI was used and erroneous in 4.77 %. In the second group without MEI, 99 patients (83.19 %) had proper localization and 16.81 % incorrect (*p* = 0.00197) (Table 2).

**Table 2:** General concordance between surgical location and preoperative endoscopic localization in both groups of patients

Group	*N*	Localization	*N*	%
I	127	Correct	121	95.28
		Incorrect	6	4.72
II	119	Correct	99	83.19
		Incorrect	20	16.81

*p* = 0.00197

The regional distribution of neoplasms was as follows: cecum (*n* = 19), ascending colon (*n* = 42), hepatic flexure (*n* = 19), transverse colon (*n* = 31), splenic flexure (*n* = 10), descending colon (*n* = 35) and sigmoid colon (*n* = 98). The percentage of erroneous range of surgical resection was significantly higher in the second group (without MEI), as presented in Table 3.

**Table 3:** Concordance between accurate surgical location and endoscopic in both groups of patients

Endoscopic location	Group	*N* consistent	*n* inconsistent	% inconsistent	Location inconsistent	
Cecum	I	6	0	0		
	II	10	3	23.1	Ascending colon Proximal 1/3	
Ascending colon Proximal 1/3	I	10	0	0		
	II	9	0	0		
Ascending colon Middle 1/3	I	9	0	0		
	II	6	0	0		
Ascending colon Distal 1/3	I	3	0	0		
	II	5	0	0		
Hepatic flexure	I	11	0	0		
	II	8	0	0		
Transverse colon Proximal 1/3	I	4	0	0		
	II	7	0	0		
Transverse colon Middle 1/3	I	7	2	22.2	Transverse colon Distal 1/3	
	II	2	1	33.3	Hepatic flexure	
Transverse colon Distal 1/3	I	3	0	0		
Splenic flexure	II	4	1	20	Splenic flexure	
	I	5	0	0		
	II	4	1	20	Descending colon Proximal 1/3	
Descending colon Proximal 1/3	I	4	0			
	II	2	1	33.3	Descending colon Middle 1/3	
Descending colon Middle 1/3	I	9	0			
	II	1	1	50	Splenic flexure	
Descending colon Distal 1/3	I	9				
	II	5	3	60	Descending colon Middle 1/3	1
					Sigmoid colon	2
Sigmoid colon >30 cm from the anal verge	I	41	4	8.9	Descending colon Middle 1/3	1
					Descending colon Distal 1/3	2
					Rectum and sigmoid colon <30 cm	1
	II	36	17	32.1	Descending colon Proximal 1/3	1
					Descending colon Middle 1/3	2
					Rectum and sigmoid colon <30 cm	14

The greatest discrepancy in localization occurred in 8.9 % of patients from group I and 32 % of patients from group II in whom the lesion position was assessed initially in sigmoid colon.

We also compared the extent to which the incorrect location influenced the change of intraoperative tactics (Table 4).

**Table 4:** Concordance of endoscopic tumor location and elective surgical procedure

Group	Region	Procedure	*n*	%
I	Right hemicolon	Right hemicolectomy	43	100
	Transverse colon	Left hemicolectomy	2	22.2
		Transversectomy	7	77.8
	Left hemicolon	Left hemicolectomy	30	100
	Sigmoid colon	Sigmoid resection	42	93.3
		Left hemicolectomy	3	6.7
II	Right hemicolon	Right hemicolectomy	48	100
	Transverse colon	Right hemicolectomy	1	33.3
		Transversectomy	2	67.6
	Left hemicolon	Sigmoid resection	2	8.7
		Left hemicolectomy	21	91.3
	Sigmoid colon	Sigmoid resection	42	93.3
		Left hemicolectomy	3	6.7

We analyzed other factors that may affect the accuracy of tumor localization, i.e., the gender, age, height, weight and BMI of patients in both groups. There was no statistical indication that any of the above parameters could affect erroneous endoscopic location changes. The statistical significance was *p* = 0.439 for age, *p* = 0.72 for sex, *p* = 0.099 for height, *p* = 0.355 for weight and *p* = 0.897 for BMI.

In this study, we did not analyze the course of the surgical procedure, the rates of conversion from laparoscopic to open or the change in intraoperative tactics as the aforementioned issues are dependent on the advancement stage of the tumor and the clinical condition of the patient.

## Discussion

The coincidence in time of widespread laparoscopic surgery (with the limitation it entails for manual colonic examination) with the foreseeable trend toward smaller tumors at the time of diagnosis confirms the need to improve the accuracy of laparoscopic colon tumor localization. Furthermore, accurate tumor localization is critical to performing minimally invasive colonic resection. The need for accurate preoperative localization of the tumor has triggered the development of different endoscopic techniques to facilitate further tumor identification at the time of surgery, including the use of clips [3–5] and peritumoral submucosal tattooing [6–9]. More recently, the use of new technologies such as the “Scope Guide” or “magnetic endoscopic imaging” has been proposed to identify the position of the endoscope in the colon [10–12], thereby facilitating lesion location detection. The electromagnetic imaging system has been introduced as an aid to colonoscopy and reveals a great potential for assisting endoscopists without exposing patients or medical staff to radiation. In our study, the endoscopic accuracy with use of MEI for colonic cancer localization was very high and significantly better than that of conventional endoscopic accuracy. Moreover, the use of MEI can shorten examination time, diminish pain on insertion and does not evoke any inconvenience for the examination to proceed [13]. Obstructive tumors and those located in the descending colon or cecum were associated with a significant increase in the risk of endoscopic localization errors.

Erroneous tumor localization can have consequences, causing a change in the planned surgical strategy, including reconversion of laparoscopic to open surgery, in 4–12 % of such cases [14, 15]. Several publications have shown that mistaken localization has been responsible for serious situations such as resecting a colonic segment that does not contain the tumor [16–18].

Colonoscopy is highly sensitive for detecting colorectal tumors, but it is associated with a considerable incidence of erroneous localization. Vignati et al. [19] reported a 14 % error rate for preoperative endoscopic localization, which led to difficulty with intraoperative localization in 4.8 % of the cases, which was mainly due to nonpalpable lesions. Piscatelli et al. [15] reported that colonoscopy had a considerable error rate (21 %) for localizing colorectal cancer, especially when previous colorectal procedures had been performed. Barium enema and CT colonography are also of great value for localizing tumors. Although barium enema is a good method for localizing exophytic and stenosing lesions, it is less effective for localizing early or flat tumors [20, 21]. In cases where a polyp has already been removed, the barium enema may not be helpful for lesion localization. In these instances, preoperative endoscopic tattooing or intraoperative colonoscopy can be performed. Computed tomography colonography is useful for detecting not only the primary tumor but also synchronous colon lesions, and it provides additional information regarding regional and distant metastatic disease, the depth of wall invasion, and the precise location of the lesion in the colon prior to surgery [20].

Magnetic endoscopic imaging is a nonradiographic imaging technique that has been developed in recent years that is capable of displaying 3D images of the scope shaft and tip within the abdominal cavity. The real-time magnetic imaging system is safe and beneficial in accurate preoperative localizing of colonic tumors compared to standard colonoscopy with no visualization, as well as improving the cecal intubation rate. However, only a few studies have reported the advantages of MEI because it is a new technique, our own research material is huge but we realize that further studies need to be performed to confirm its role for planning of the extension of laparoscopic colon resection.

## Conclusions

A magnetic positioning system for the endoscope allows the more accurate localization of neoplastic infiltrate within the large intestine compared to standard colonoscopy alone, especially within the sigmoid colon. This method can be particularly useful in planning and performing a laparoscopic procedure to diminish the likelihood of improper bowel segment resection.

## Electronic supplementary material

Below is the link to the electronic supplementary material.

Video 1 Real-time, three-dimensional Scope Guide image of the shape and configuration of the colonoscope during a cecal intubation (MP4 6123 kb)
